# Establishment of the early gut microbiota in vaginally delivered infants: the influence of maternal gut microbiota outweighs vaginal microbiota

**DOI:** 10.1128/spectrum.01775-25

**Published:** 2025-08-12

**Authors:** Haishan Xie, Lulu Meng, Xia Duan, Xinyuan Liang, Ting Huang, Guangyu Ma, Huijuan Luo, Xiaomei Tang, Xiaomin Xiao

**Affiliations:** 1 Department of Obstetrics and Gynecology, First Affiliated Hospital of Jinan University 162698, Guangzhou, China; 2 Department of Obstetrics and Gynecology, Guangzhou Women and Children’s Medical Center, Guangzhou, China; 3 Department of Obstetrics, Shenzhen Maternity & Child Healthcare Hospital, The First School of Clinical Medicine, Southern Medical University 248258, Shenzhen, China; 4 Department of Obstetrics, Guangdong Women and Children Hospital 90405 , Guangzhou, China; Cleveland Clinic Lerner Research Institute, Cleveland, Ohio, USA

**Keywords:** infant, gut microbiota, microbial transmission, correlation network, 16S rDNA

## Abstract

Gut microbiota is one of the largest microbial communities within the human body, yet its origins and early developmental processes remain not fully understood. This study collected samples from 26 mother-infant pairs from the third trimester to 14 days postpartum, using 16S rDNA sequencing and Source Tracker analysis to investigate the dynamic changes and sources of early infant gut microbiota. Results showed that maternal gut microbiota at 32 weeks of gestation was the primary source of meconium microbiota in vaginally delivered infants and continued to influence day-14 gut microbiota, while maternal vaginal microbiota contributed minimally (<1%). Regardless of bacterial presence in breast milk, maternal gut microbiota remained a critical source for day-14 gut microbiota of vaginally delivered infants. Placental microbiota was also an important source of meconium microbiota across different delivery modes. In cesarean-delivered infants, the origins of gut microbiota were more complex, with a higher proportion of “transient bacteria” in meconium, potentially impacting the stable colonization of gut microbiota. However, by day 14, the gut microbiota of cesarean and vaginally delivered infants became similar post-breastfeeding. Correlation network analysis revealed intricate maternal-infant microbial interactions, indicating that maternal microbes may influence the colonization of infant gut microbiota. Shared taxa analysis showed that functional flora might exist in the placenta. Meanwhile, beneficial anaerobes like *Bifidobacterium* significantly increased in infant day-14 gut microbiota, suggesting their potential role in gut health. Overall, this study provides novel insights into the colonization and developmental patterns of early infant gut microbiota.

**IMPORTANCE:**

Gut microbiota exerts a significant impact on an individual's long-term health; however, its origins and colonization processes remain to be fully elucidated. This study revealed that early infant gut microbiota of vaginally delivered infants primarily derived from maternal gut microbiota, which began colonizing the fetus as early as 32 weeks of gestation. In contrast, the contribution of maternal vaginal microbiota to early infant gut microbiota was quite limited. Moreover, placental microbiota also constituted an important source for the fetal gut microbiota. These findings provide novel insights into the developmental mechanisms of infant gut microbiota and highlight the important role of maternal microbes in the early colonization of infant gut microbiota.

## INTRODUCTION

The human body harbors a diverse microbial community that plays a crucial role in maintaining overall health. Among these, the intestinal microbiota, comprizing bacteria, fungi, archaea, viruses, and parasites (1), is particularly significant due to its involvement in metabolism, immunity, and nervous system regulation (2, 3). The colonization of the gut microbiome begins early in life and significantly influences long-term health outcomes (4), with disruptions in infancy potentially leading to increased risks of allergic (5, 6) and metabolic diseases later in life (7, 8).

While the traditional view held that the fetal environment is sterile (9), emerging evidence suggests the possibility of microbial transmission *in utero* (10, 11). Factors such as delivery mode, maternal health, gestational age, feeding practices, and environmental influences shape the infant gut microbiota (10, 12, 13). The practice of “vaginal seeding” has garnered attention as a potential intervention for cesarean-delivered infants, although its health benefits remain uncertain (14, 15).

Although previous studies have indicated that maternal microbial reservoirs impact the establishment of fetal and infant gut microbiota (10, 11, 16, 17), few studies have systematically explored the influence of the extensive maternal microbiome on the establishment of offspring gut microbiota. Moreover, 16S rDNA sequencing is widely used for microbial community composition analysis (18, 19), and Source Tracker analysis has been proven to be effective in tracking microbial source relationships (20, 21). Consequently, based on the above-mentioned 16S rDNA sequencing and Source Tracker analysis methods, this study aims to investigate the patterns of microbial mother-to-infant transmission and the developmental regularities of early infant gut microbiota, contributing to a better understanding of the formation mechanism of the infant gut microbiome and providing theoretical support for improving infant health care strategies.

## RESULTS

### Study population

A total of 26 mother-infant pairs were enrolled in the study, comprizing 17 vaginal deliveries and 9 cesarean deliveries. There were no significant differences in maternal characteristics such as age, height, weight, BMI, and gestational age at delivery, nor in infant characteristics including birth weight, length, head circumference, 1-min Apgar score, and gender between the two delivery groups. Detailed clinical characteristics of the participants are summarized in Table 1. Participants were further categorized into four groups based on the sample collection process: meconium microbiota of vaginally delivered infants (VIF1, *n* = 8), gut microbiota of 14-day-old vaginally delivered infants (VIF2, *n* = 16), meconium microbiota of cesarean-section infants (CIF1, *n* = 7), and gut microbiota of 14-day-old infants delivered by cesarean section (CIF2, *n* = 3).

**TABLE 1:** Clinical characteristics of mother-infant pairs

	Vaginal delivery (*n* = 17)	Cesarean delivery (*n* = 9)	*P*
Maternal age (years)	26.6 ± 0.49	30.6 ± 1.91	0.075
Height (m)	1.60 ± 1.63	1.60 ± 1.11	0.844
Weight (kg)	65.7 ± 2.31	64.6 ± 1.93	0.701
Gestational BMI (kg/m^2^)	25.5 ± 0.52	25.3 ± 0.80	0.834
Gestational week (weeks)	39.2 ± 0.26	39 ± 0.27	0.534
Birth height (cm)	49.4 ± 0.32	49.1 ± 0.42	0.597
Birth weight (g)	3,161.8 ± 63.96	3,072.2 ± 141.94	0.513
Birth head circumference (cm)	33.4 ± 0.17	33.2 ± 0.49	0.367
Apgar score (1 min)	9 ± 0.06	9 ± 0.11	0.833
Infants’ gender (*n*, %)			1
Male	11 (64.7%)	6 (66.7%)	
Female	6 (35.3%)	3 (33.3%)	

### Diversity and composition of gut microbiota during early infancy

**Fig 1:**
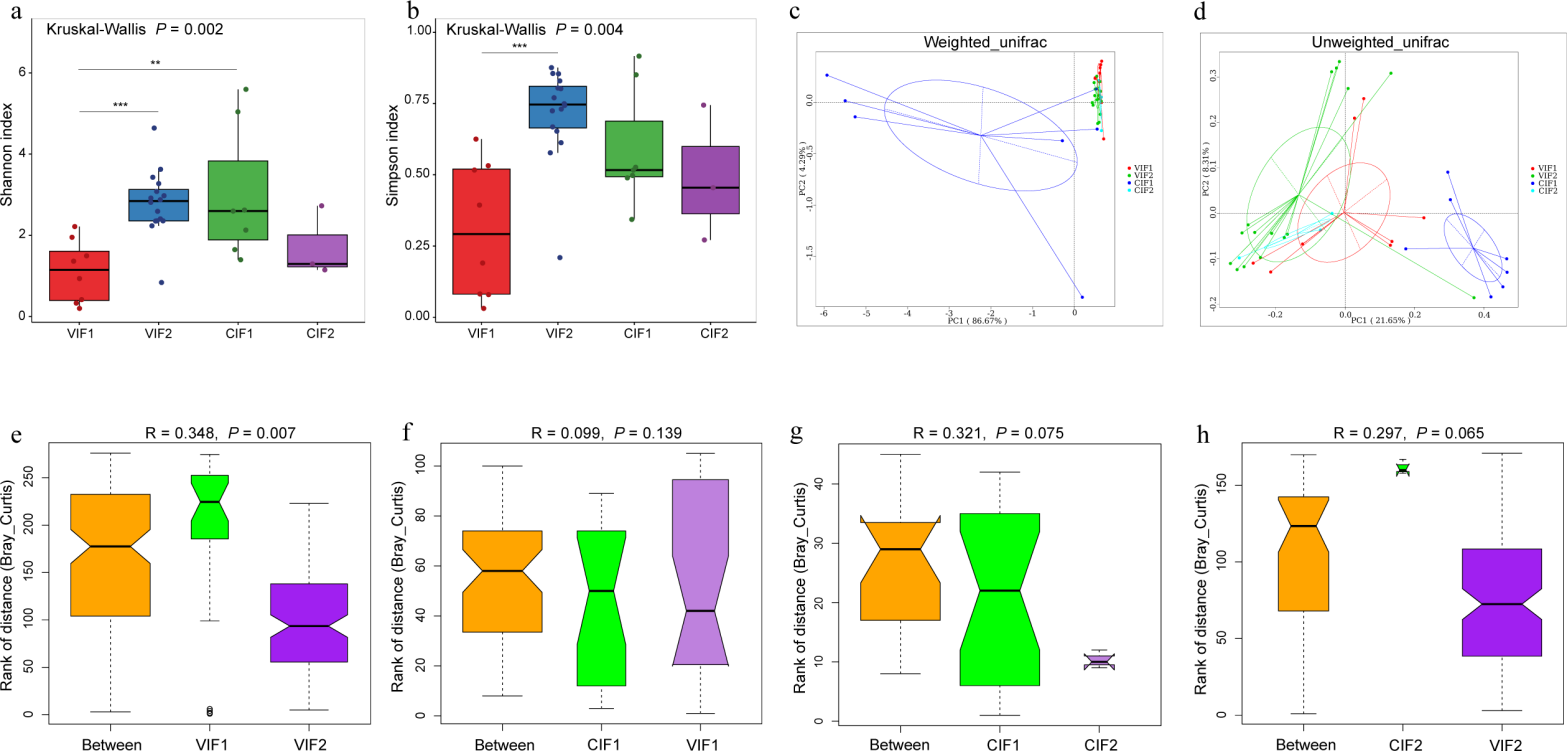
α and β diversity analyses of early infant gut microbiota. (**a**) Shannon index and (**b**) Simpson index were utilized to assess α diversity. Group differences were tested using the Kruskal-Wallis test, with pairwise comparisons conducted using the Wilcoxon rank-sum test with Bonferroni correction for multiple testing. Statistical significance is indicated by **P < 0.01 and ***P < 0.001. (**c**) Principal coordinates analysis (PCoA) based on Weighted_Unifrac distances and (**d**) Unweighted_Unifrac distances was performed to visualize the clustering of samples. Different colors represent distinct groups, and the percentages on the axes indicate the proportion of variance explained by each principal component. (**e–h**) Anosim analysis based on Bray-Curtis dissimilarity was performed to evaluate the differences in microbial community structure between groups at different time points. CIF1, meconium microbiota of cesarean-section infants; CIF2, gut microbiota of 14-day-old infants delivered by cesarean section; VIF1, meconium microbiota of vaginally delivered infants; VIF2, gut microbiota of 14-day-old vaginally delivered infants.

The α diversity analysis revealed significant variations in infant gut microbiota across different stages. The VIF2 exhibited the highest richness and evenness, while the VIF1 showed the lowest richness and evenness levels (Fig. 1a and b). Additionally, both VIF2 and CIF1 demonstrated significantly higher Shannon index values compared to VIF1 (*P* < 0.001 and *P* < 0.01, respectively, Fig. 1a). The Simpson index was also notably higher in VIF2 compared to VIF1 (*P* < 0.001, Fig. 1b). Principal coordinates analysis (PCoA) visualized the β diversity of gut microbiota in infants across different delivery modes. While the clustering of cesarean-delivered infants' meconium samples was not significant, this indicated considerable intra-group variance. In contrast, the other three groups showed smaller differences in gut microbial composition, with substantial overlap among samples (Fig. 1c and d). ANOSIM analysis confirmed significant differences in early gut microbial community structure among infants born by different delivery modes (*R* = 0.465, *P* = 0.001). Notably, only meconium microbiota and day-14 gut microbiota of vaginally delivered infants showed significant differences (*R* = 0.348, *P* = 0.007), with no significant disparities among the other groups (Fig. 1e through h). These findings emphasize the impact of delivery mode and developmental timing on infant gut microbiota diversity.

**Fig 2:**
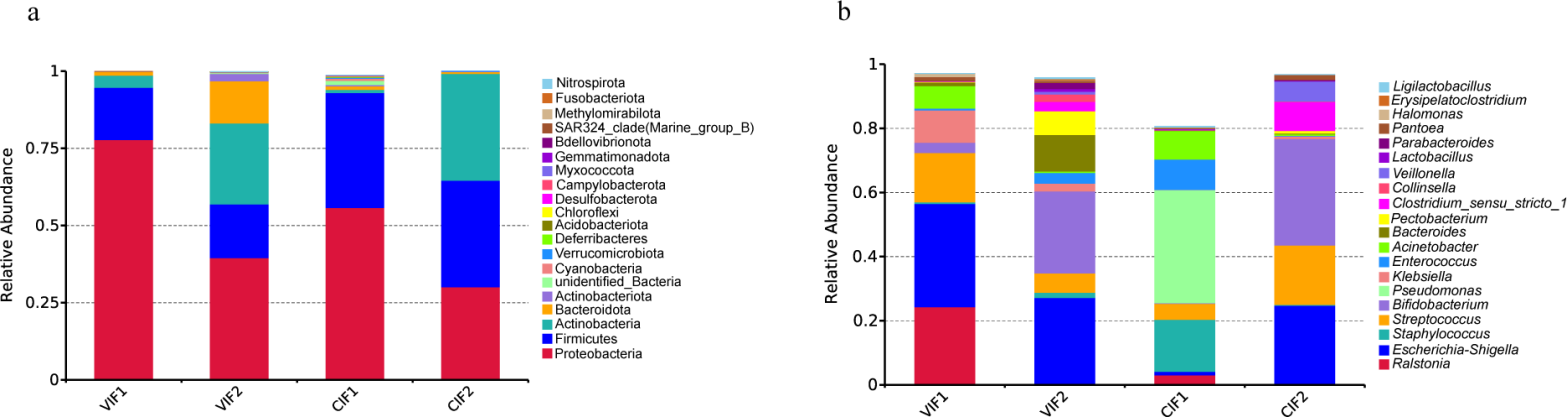
Relative abundances of the top 20 microbes in early infant gut microbiota at the phylum and genus levels. (**a**) Phylum-level composition of the top 20 microbes ranked by relative abundance in meconium and day-14 gut microbiota of infants. (**b**) Genus-level composition of the top 20 microbes ranked by relative abundance in meconium and day-14 gut microbiota of infants. CIF1, meconium microbiota of cesarean-section infants; CIF2, gut microbiota of 14-day-old infants delivered by cesarean section; VIF1, meconium microbiota of vaginally delivered infants; VIF2, gut microbiota of 14-day-old vaginally delivered infants.

**Fig 3:**
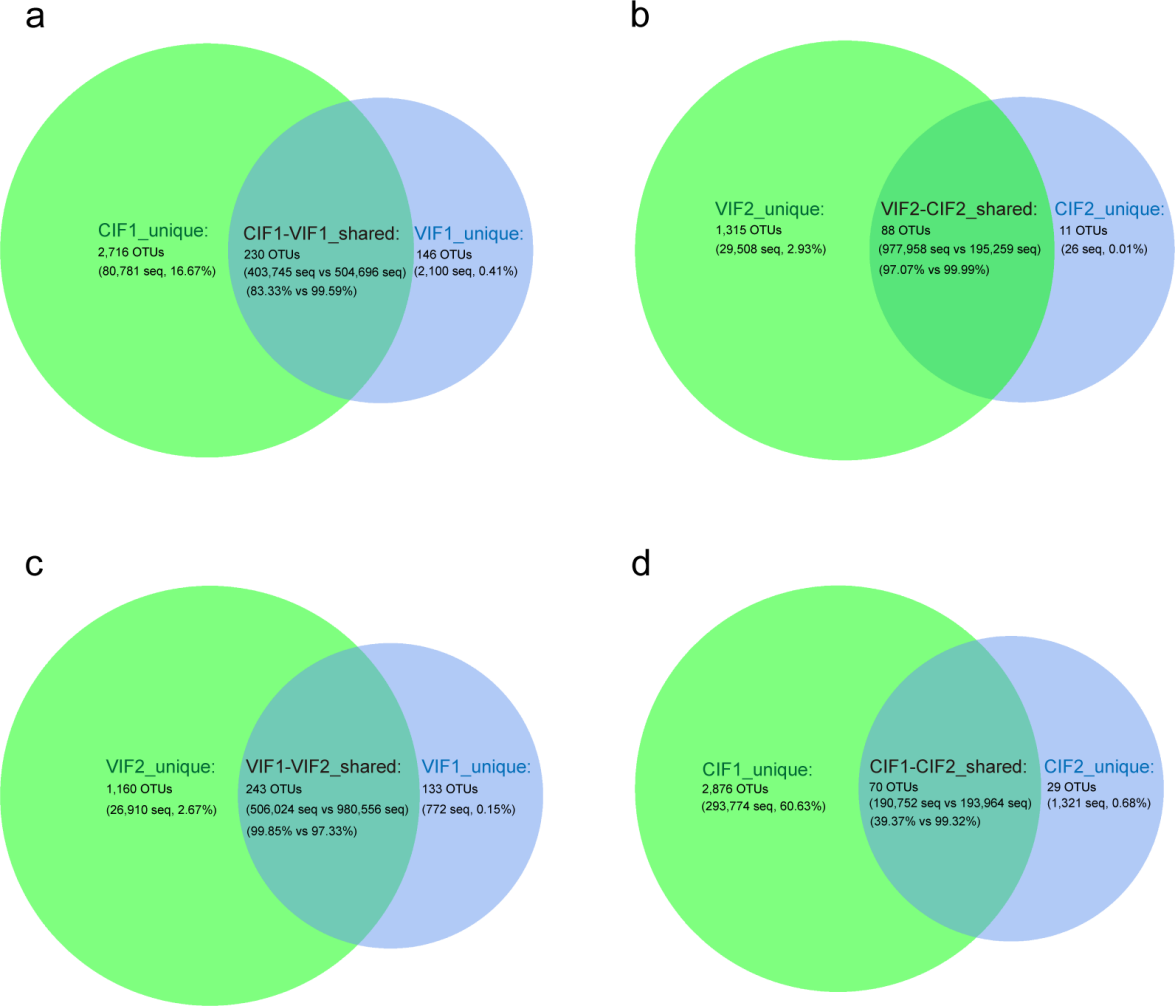
Venn diagrams of early infant gut microbiota. (**a**) Venn diagram showing the unique and shared OTU counts, sequence numbers (seq), and relative abundances between the meconium microbiota of vaginally delivered (VIF1) and cesarean-section infants (CIF1). (**b**) Venn diagram depicting unique and shared OTU counts, seq, and relative abundances between VIF2 and CIF2. (**c**) Venn diagram presenting shared and unique OTU counts, seq, and relative abundances between VIF1 and VIF2. (**d**) Venn diagram illustrating shared and unique OTU counts, seq, and relative abundances between CIF1 and CIF2. In each diagram, overlapping areas represent shared OTUs, while non-overlapping areas indicate unique OTUs, along with corresponding sequence counts and relative abundances. Relative abundances, expressed as percentages, were calculated by dividing the sequence counts of unique or shared OTUs by the total OTU sequence count within each group and multiplying by 100% to adjust for sample size discrepancies between groups. OTU, operational taxonomic unit; seq, sequences.

To characterize the gut microbiota during early infancy, we analyzed the composition at the phylum, genus, and operational taxonomic unit (OTU) levels. At the phylum level, Proteobacteria, Firmicutes, Actinobacteria, and Bacteroidota were dominant from the meconium to day-14 gut microbiota, regardless of delivery mode. Our findings revealed that the relative abundance of Proteobacteria decreased and Actinobacteria increased significantly during this transition, while the level of Firmicutes remained stable. Interestingly, the relative abundance of Bacteroidota increased significantly over time in vaginally delivered infants, while remaining consistently low in those delivered by cesarean section (Fig. 2a; Table S2). At the genus level, *Escherichia-Shigella* (32.22%), *Ralstonia* (24.33%), *Streptococcus* (15.40%), and *Klebsiella* (10.09%) were predominant in VIF1. By day 14, *Escherichia-Shigella* (27.21%), *Bifidobacterium* (25.54%), and *Bacteroides* (11.39%) dominated in VIF2. In contrast, *Pseudomonas* (35.31%), *Staphylococcus* (16.23%), and *Enterococcus* (9.48%) were prevalent in CIF1, and by day 14, *Bifidobacterium* (33.25%), *Escherichia-Shigella* (24.82%) and *Streptococcus* (18.53%) were dominant in gut microbiota of cesarean-section infants (Fig. 2b; Table S3). Venn diagrams integrating OTU counts, sequence numbers, and relative abundances revealed distinct microbial community overlaps between vaginally and cesarean-delivered infants. In meconium microbiota, 230 (7.44%) shared OTUs accounted for 99.59% (504,696 sequences) of sequences in vaginally delivered infants and 83.33% (403,745 sequences) in cesarean-delivered infants (Fig. 3a). By day 14, shared OTUs decreased to 88 (6.22%), but these OTUs accounted for the majority of sequence numbers in both groups (VIF2: 97.07%, CIF2: 99.99%), suggesting that these shared OTUs could be key components of the infant gut microbiota at day 14 (Fig. 3b). Longitudinal analysis showed that the number of OTUs increased over time from meconium to day-14 gut microbiota in vaginally delivered infants, with a total of 243 (15.82%) OTUs shared between the two time points, comprizing 99.85% (506,024 sequences) of meconium sequences and 97.33% (980,556 sequences) of day-14 sequences (Fig. 3c). In contrast, the number of OTUs decreased over time from meconium to day-14 gut microbiota in cesarean-delivered infants, with only 70 (2.35%) OTUs shared between the two time points, accounting for 39.37% (190,752 sequences) of meconium sequences and 99.32% (193,964 sequences) of day-14 sequences, indicating a significant shift in microbial composition (Fig. 3d). These findings illustrate that delivery mode may influence the trajectory of microbial community succession in early infant gut microbiota.

**Fig 4:**
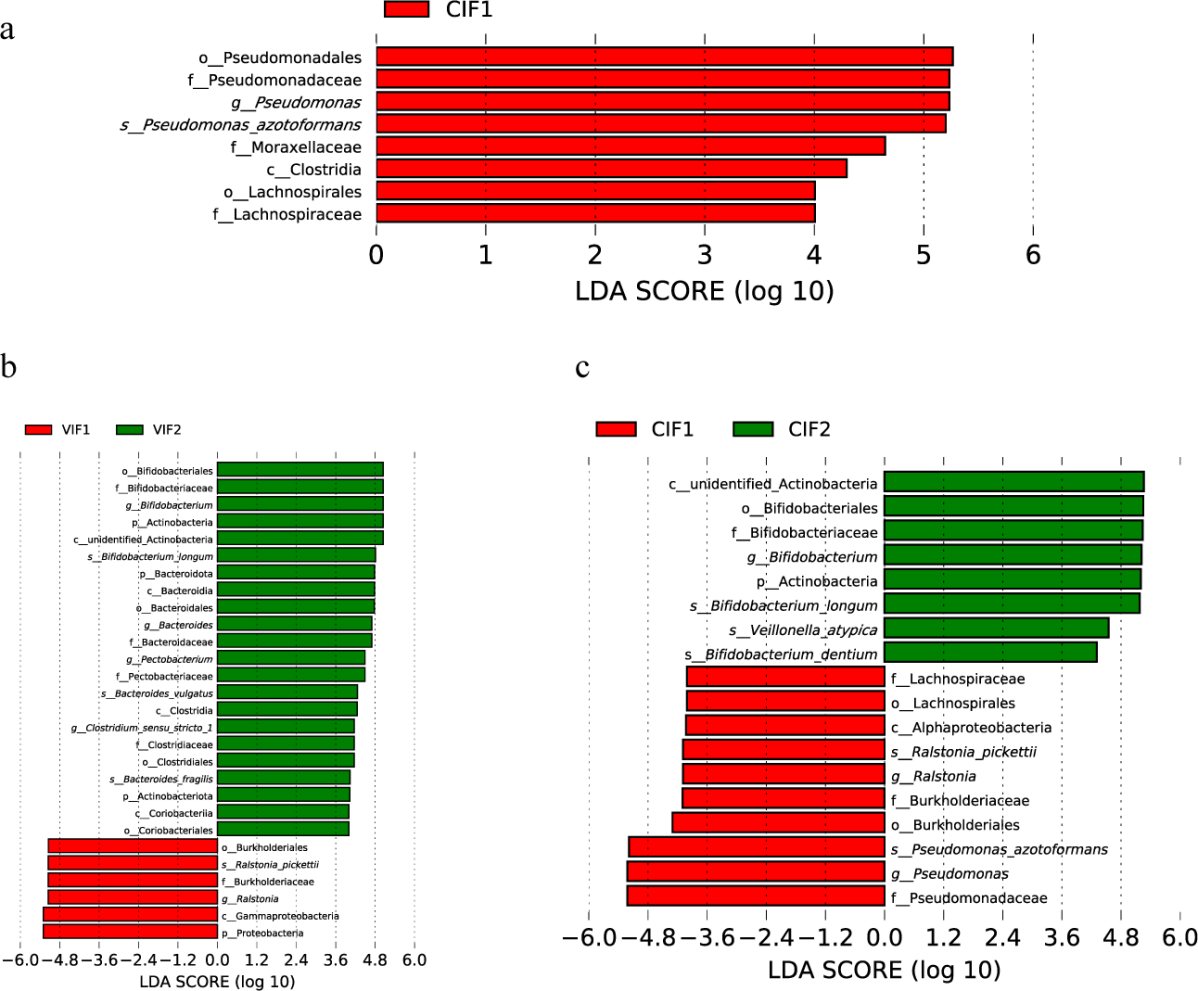
LEfSe analysis of gut microbiota during early infancy. (**a**) LEfSe analysis revealed distinct differences in composition between the meconium microbiota of vaginally delivered infants (VIF1) and that of cesarean-section infants (CIF1). (**b**) Significant variations were observed in composition between the VIF1 and the gut microbiota of 14-day-old vaginally delivered infants (VIF2). (**c**) Notable disparities in composition were indicated between the CIF1 and the gut microbiota of 14-day-old infants delivered by cesarean section (CIF2). LEfSe, Linear discriminant analysis effect size.

Linear discriminant analysis effect size (LEfSe) was performed to identify taxa with significant differences in infants' gut microbiota by different modes. In vaginally delivered infants, Proteobacteria were significantly enriched in meconium microbiota, whereas Actinobacteria and Bacteroidota were enriched in day-14 gut microbiota. At the genus level, *Ralstonia* was predominant in meconium microbiota, and *Bacteroides*, *Bifidobacterium*, *Clostridium_sensu_stricto_1*, and *Pectobacterium* were dominant in day-14 gut microbiota. At the species level, *Ralstonia pickettii* was enriched in meconium, while *Bifidobacterium longum*, *Bacteroides vulgatus,* and *Bacteroides fragilis* were more abundant in day-14 gut microbiota (Fig. 4b). In cesarean-section infants, Actinobacteria were enriched at the phylum level in day-14 gut microbiota. *Ralstonia* and *Pseudomonas* were more abundant in meconium, and *Bifidobacterium* was enriched in day-14 gut microbiota. At the species level, *R. pickettii* and *Pseudomonas azotoformans* were enriched in meconium microbiota, while *B. longum* and *Bifidobacterium dentium* were more abundant in day-14 gut microbiota (Fig. 4c). Moreover, *Pseudomonas* was more prevalent in meconium microbiota of cesarean infants compared to vaginally born infants (Fig. 4a). No significant differences were found between 14-day-old infants’ gut microbiota in VIF2 and CIF2 when LDA was set to 4.

### Maternal microbiota contributions to early infant gut microbiota

**Fig 5:**
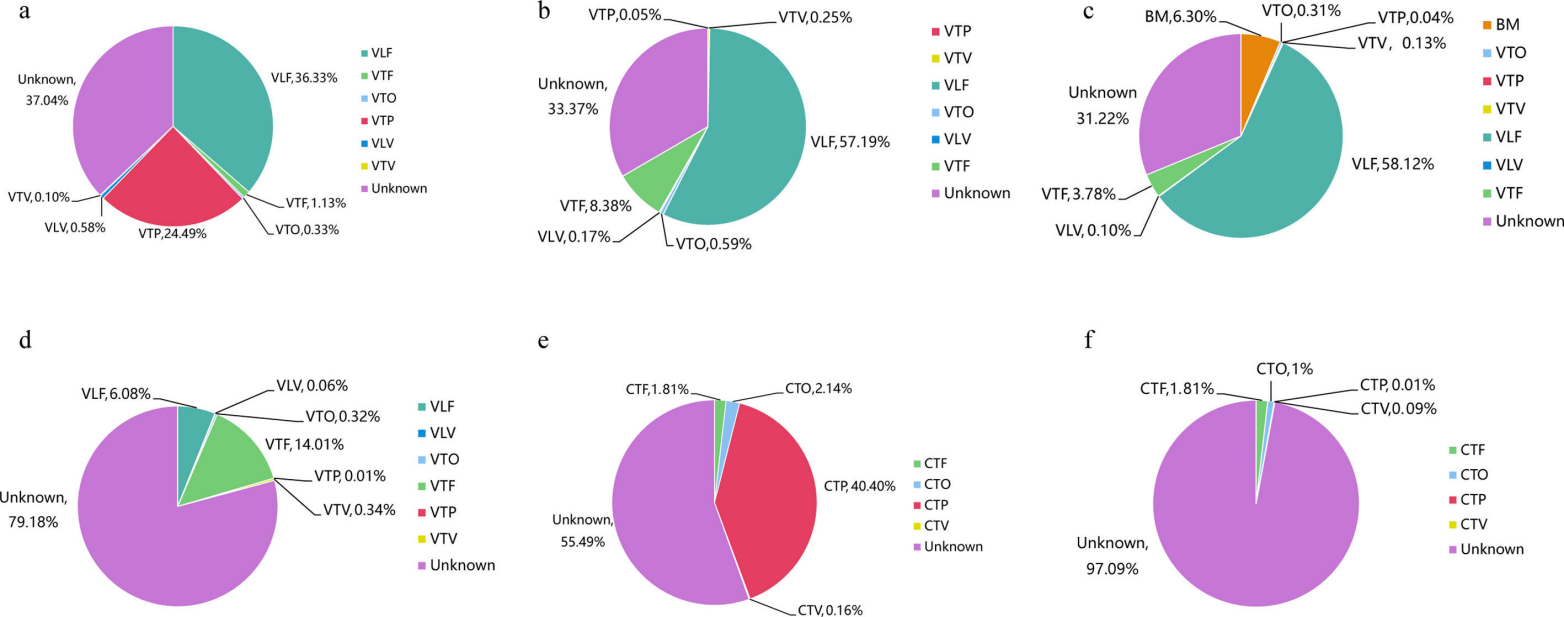
Contributions of maternal niche microbiota on the early infant gut microbiota. Results of Source Tracker analysis for the following groups: (**a**) meconium microbiota of vaginally delivered infants (VIF1, *n* = 8), (**b**) gut microbiota of 14-day-old vaginally delivered infants (VIF2, *n* = 16), (**c**) gut microbiota of 14-day-old vaginally delivered infants fed bacterial breast milk (BM, *n* = 10), (**d**) gut microbiota of 14-day-old vaginally delivered infants fed sterile BM (*n* = 4), (**e**) meconium microbiota of cesarean-section infants (CIF1, *n* = 7), and (**f**) gut microbiota of 14-day-old infants delivered by cesarean section (CIF2, *n* = 3). CTF, gut microbiota at term pregnancy in women with cesarean section delivery; CTO, maternal oral microbiota at term in women with cesarean section delivery; CTP, full-term placental microbiota of cesarean section; CTV, vaginal microbiota at term in women with cesarean section delivery; VLF, gut microbiota at 32 weeks in women with vaginal delivery; VLV, vaginal microbiota at 32 weeks of gestation in women with vaginal delivery; VTF, gut microbiota at term pregnancy in women with vaginal delivery; VTO, maternal oral microbiota at term in women with vaginal delivery; VTP, full-term placental microbiota of vaginal delivery; VTV, vaginal microbiota at term in women with vaginal delivery.

To investigate the contribution of maternal microbiota to early infant gut microbiota, we employed Source Tracker analysis. For infants delivered vaginally, the overall contribution of the maternal microbiota to infant gut microbiota remained stable. However, the relative contributions from different maternal niches varied. For meconium microbiota, maternal gut microbiota at 32 weeks (VLF) and full-term placental microbiota (VTP) were the main sources, contributing 36.33% and 24.49%, respectively (Fig. 5a). By day 14, the contribution of VLF surged to 57.19%, with maternal gut microbiota at term pregnancy (VTF) becoming the second largest contributor at 8.38%. Additionally, the contribution of VTP dropped sharply to 0.05% (Fig. 5b). Other maternal niches, such as maternal oral microbiota at term pregnancy (VTO), maternal vaginal microbiota at 32 weeks (VLV), and maternal vaginal microbiota at term pregnancy (VTV), had minimal impact on early infant gut colonization, each contributing less than 1% (Fig. 5a and b).

To assess the contribution of breast milk (BM) microbiota to infant gut microbiota, we collected 14-day postpartum BM samples from mothers who had vaginal deliveries. For 14-day-old vaginally delivered infants fed with bacterial BM, Source Tracker analysis showed that the maternal microbiota accounted for 68.78% of infant gut microbiota colonization, with the largest contribution from VLF at 58.12%, followed by BM microbiota at 6.30%. Other sources had minimal contributions, and microorganisms of unknown origin accounted for 31.22% (Fig. 5c). Significant differences in the contributions of maternal microbiota from various niches to these infants' day-14 gut microbiota were observed (*P* < 0.001). In contrast, for infants fed with sterile BM (not effectively sequenced due to very low bacteria or no detectable bacteria), the maternal microbiota contributed 20.82% to the day-14 infant gut microbiota. Among them, the maternal gut microbiota at term pregnancy (VTF) had the highest contribution at 14.01%, and unknown microorganisms accounted for 79.18% (Fig. 5d). Significant differences in the maternal microbiota contributions to the day-14 gut microbiota of infants fed with sterile BM were also detected (*P* < 0.001).

To explore whether delivery mode influences maternal contributions to infant gut microbiota, we traced origins of gut microbes in cesarean-section infants. The full-term placental microbiota (CTP) contributed the most to the meconium microbial community (40.40%). By day 14, maternal influence decreased significantly, with the maternal full-term gut microbiota (CTF) contributing the most at a stable 1.81%, while the contribution of CTP dropped to only 0.01%. The maternal full-term oral microbiota (CTO) contributed 2.14% to the meconium microbiota and 1% to the infant day-14 gut microbiota. Meanwhile, the maternal full-term vaginal microbiota (CTV) accounted for only 0.16% and 0.09%, respectively (Fig. 5e and f).

### Cross-ecological and temporal interactions between maternal and infant microbiota

To further explore the impact of maternal microbiota on early infant gut microbiota, especially its potential influence on *Bifidobacterium* colonization, we constructed correlation networks to visualize microbial interactions across different time points and ecological niches. These networks unveil the complex associations between the infant gut microbiota at various stages and the maternal microbiota that makes a relatively significant contribution to it.

**Fig 6:**
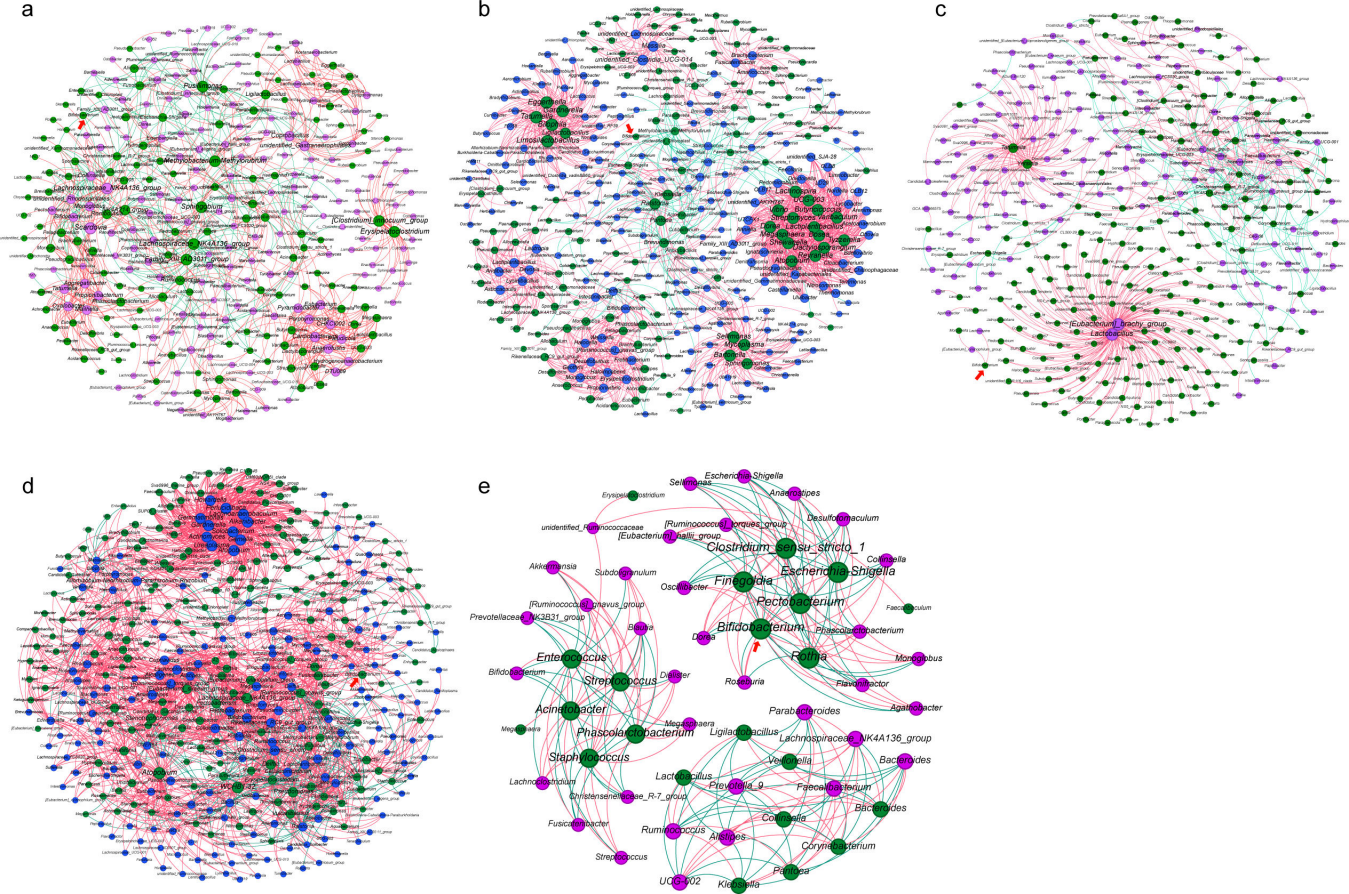
Correlation networks of cross-ecological and temporal interactions between maternal and infant microbiota. Correlation networks were constructed at the genus level for the following pairs: (**a**) meconium microbiota of vaginally delivered infants (VIF1) and maternal gut microbiota at 32 weeks (VLF); (**b**) VIF1 and full-term placental microbiota of vaginal delivery (VTP); (**c**) gut microbiota of 14-day-old vaginally delivered infants (VIF2) and VLF; (**d**) meconium microbiota of cesarean-section infants (CIF1) and full-term placental microbiota of cesarean section (CTP); (**e**) gut microbiota of 14-day-old infants delivered by cesarean section (CIF2) and full-term gut microbiota of women with cesarean section delivery (CTF). In the network diagrams, green dots denote infant gut microbiota, purple dots represent maternal gut microbiota, and blue dots indicate placental microbiota. Red arrows indicate the location of *Bifidobacterium* to highlight its associations with other genera in the network diagrams. Red lines signify positive correlations, while green lines indicate negative correlations. For each network, the top 200 genera based on relative abundance were selected from each microbial niche. However, in the CIF2-CTF network, only 35 genera were detected in CIF2; therefore, the top 35 genera by relative abundance from CTF were selected for analysis.

For vaginally delivered infants, in the VIF1-VLF correlation network, low-abundance genera such as *Clostridium innocuum group* in VIF1 and *Lachnospiraceae_NK4A136_group* in the VLF occupied central positions (Fig. 6a; Table S4). Six genera in VLF were significantly correlated with *Bifidobacterium* in VIF1. Among them, *Lachnospiraceae_NK4A136_group* showed a positive correlation, while *Bifidobacterium* in VLF was negatively correlated with *Bifidobacterium* in VIF1 (Table S5), suggesting the VLF may influence infant intestinal *Bifidobacterium* colonization. Additionally, in the VTP-VIF1 correlation network, low-abundance genera such as *Lautropia* in VTP and *Lachnospira* in VIF1 were at the core (Fig. 6b; Table S4). Three genera in VTP were strongly positively correlated with *Bifidobacterium* in VIF1, indicating that placental microbes may play a certain role in intestinal *Bifidobacterium* colonization in early infancy (Table S6). In the VIF2-VLF correlation network, low-abundance genera like *Tatumella* in the infant day-14 gut microbiota and *Lactobacillus* in VLF also occupied central positions (Fig. 6c; Table S7). Additionally, *[Eubacterium]_xylanophilum_group* and *Lachnospira* in VLF were positively correlated with *Bifidobacterium* in VIF2, indicating their potential impact on shaping infant intestinal microecology (Table S8).

For cesarean-section infants, the correlation network between CTP and CIF1 revealed that low-abundance genera, such as *Atopobium* in CIF1 and *Gemella* in CTP, played central roles in forming a tightly connected network, with most correlations being positive (Fig. 6d; Table S9). Notably, 20 genera in CTP were associated with *Bifidobacterium* in CIF1, 18 of which showed positive correlations (Table S10). In the correlation network between CIF2 and CTF, since only 35 genera were identified in CIF2, we analyzed the top 35 abundant genera in CTF. The correlation network revealed three independent modules, with genera such as *Bifidobacterium* and *Escherichia-Shigella* in CIF2 occupying central positions (Fig. 6e; Table S11). Fourteen genera in CTF were correlated with *Bifidobacterium* in CIF2, with seven positive and seven negative correlations (Table S12). This may reflect the intricate regulatory influence of maternal microbiota on infant gut microbiota composition.

### Analysis of shared taxa between placenta, meconium, and early infant gut microbiota

The existence of bacteria in the placenta remains a topic of debate. To further investigate the potential contribution of the placental microbiota to the meconium microbiota, we analyzed their shared species and aerotolerance. Our results showed that 14 species with relative abundances ≥ 0.1% were shared between VTP and VIF1, with four species in VTP and 12 species in VIF1. Common human symbiotic bacteria such as *B. longum* and *Escherichia coli* were identified. Most of these species were anaerobes or facultative anaerobes (Table S13). Similarly, between CTP and CIF1, 20 shared species with relative abundances ≥ 0.1% were identified, including 16 species in CTP and 12 species in CIF1. Common symbiotic bacteria, including *Escherichia coli* and *Enterococcus faecalis,* were detected, with the majority being anaerobes or facultative anaerobes (Table S14). Notably, regardless of delivery mode, *P. azotoformans* and *R. pickettii* exhibited relatively high abundances in the placental microbiota (Tables S13 and S14).

We next analyzed shared genera between meconium microbiota and gut microbiota of 14-day-old infants to explore the colonization process of early infant gut microbiota. In vaginally delivered infants, 15 shared genera with relative abundances ≥1% between meconium microbiota and infant day-14 gut microbiota were identified, including 9 genera in meconium microbiota and 11 genera in day-14 gut microbiota. Most of these shared genera are common human symbiotic bacteria crucial for maintaining gut health. Facultative anaerobes such as *Escherichia-Shigella* maintained relatively high abundances at both stages. Notably, the abundance of *Klebsiella* decreased, the level of *Enterococcus* increased, along with the abundances of anaerobic bacteria such as *Bifidobacterium*, *Bacteroides,* and *Clostridium_sensu_stricto_1* substantially increased (Table S15). For cesarean-section infants, 10 shared genera with relative abundances ≥1% were identified between meconium and day-14 gut microbiota, comprizing 5 genera in meconium and 7 genera in day-14 gut microbiota. Over time, the abundance of *Enterococcus* declined, while the abundance of *Escherichia-Shigella* increased significantly. Additionally, the abundances of anaerobic bacteria like *Bifidobacterium*, *Clostridium_sensu_stricto_1*, and *Veillonella* also showed significant increases (Table S16).

## DISCUSSION

The colonization of early infant gut microbiota significantly impacts future health, but our understanding remains limited. This study conducted a comprehensive analysis to investigate the transmission of maternal-infant microbiota and the development of early infant gut microbiota.

Our findings indicate that the mode of delivery significantly influences infant gut microbiota development. Infants delivered vaginally exhibited increasing gut microbiota α diversity over time, while cesarean-born infants showed a declining trend, consistent with previous research reporting lower gut microbiota diversity in cesarean-born infants during the first 2 years of life (22). Additionally, cesarean-delivered infants showed a higher Firmicutes/Bacteroidetes ratio (F/B ratio), a microbial alteration associated with increased risk of obesity and other diseases (23–25). This underscores the potential long-term effects of delivery mode on infant gut microbiota composition and subsequent health outcomes. Furthermore, we observed significant enrichment of *Bifidobacterium* in day-14 infant gut microbiota, potentially associated with exclusive breastfeeding (26, 27). This is supported by previous research that highlights BM’s role in promoting the early colonization of beneficial microbes, including *Bifidobacterium*, which is essential for gut health and immune system development (28).

At the genus level, *Escherichia-Shigella* exhibited dominance in VIF1, consistent with previous study (29). In contrast, CIF1 was dominated by *Staphylococcus* and *Pseudomonas*, which are common constituents of skin microbiota and environmental samples, respectively (30, 31). The Venn diagram showed a decreasing trend in the OTU numbers of gut microbiota in cesarean-delivered infants over time, and the relative abundance of shared OTUs increased significantly (from 39.37% to 99.32%). Additionally, CIF1 exhibited higher richness and evenness compared to vaginally delivered infants, likely due to environmental influences rather than normal intestinal colonizing microbes. Consequently, as the developmental process progresses, these “transient bacteria” from the environment tend to disappear. In contrast, the relative abundance of shared OTUs remained stable from meconium to day-14 gut microbiota (99.85% vs 97.33%) in vaginally delivered infants. This stability, along with the increase in the number of OTUs over time, may be due to a higher proportion of normal gut colonizing microbes in meconium, which could promote the transition of infant gut microbiota to a more stable and mature state (32). β diversity analysis revealed significant intra-group variability in CIF1, likely reflecting the complex influences of maternal skin microorganisms and environmental factors (33, 34), which may affect the stability of infant gut colonization. Notably, by day 14, *Bifidobacterium* emerged as the dominant genus in infant gut microbiota, and no significant differences in taxa were observed between day-14 gut microbiota of vaginally and cesarean-delivered infants. This indicates that although cesarean-born infants harbor a higher proportion of “transient bacteria” in their meconium microbiota, after 14 days of breastfeeding, their gut microbiota composition becomes more similar to that of vaginally delivered infants.

The transmission of infant gut microbiota from mothers during birth is widely acknowledged (35, 36), with “microbial transmission” even being considered a form of epigenetic inheritance (37). Infants delivered vaginally are more likely to acquire maternal microbial strains, while cesarean-delivered infants may take longer (38). Our research revealed that maternal oral, intestinal, vaginal, and placental microbiota contributed to infant meconium microbiota, supporting the idea that meconium microbiota originates from diverse maternal microbial reservoirs (11, 29). Additionally, our findings indicated that VLF significantly contributed to VIF1, suggesting that maternal gut microbes may be transferred to the fetus via endogenous pathways as early as 32 weeks. Interestingly, although infants delivered vaginally are more likely to be exposed to maternal vaginal microbes, Source Tracker analysis revealed a relatively limited contribution of vaginal microbiota to both meconium and day-14 gut microbiota, consistent with previous study (39). This implies that the role of vaginal microbes in shaping meconium microbiome may be less significant than previously thought (33, 34). Consequently, our results do not provide strong evidence that “vaginal seeding” in cesarean-delivered infants can significantly restore gut microbial colonization (40). Moreover, our study demonstrated that BM microbiota is an important source for day-14 gut microbiota of vaginally delivered infants, emphasizing the essential role of breastfeeding in gut colonization (41, 42). Importantly, regardless of the presence of bacteria in BM, maternal gut microbiota remains the primary known source of infant gut microbiota. This indicates that even with contributions from BM microbes, maternal gut microbiota continues to play a vital role in early infant gut colonization.

For cesarean-delivered infants, Source Tracker analysis indicated a significant contribution of placental microbiota to CIF1, along with a relatively high abundance of environmental microbes like *Pseudomonas* (43), which may be related to environmental microbial contamination or lack of exposure to vaginal microbiota during delivery, leading to insufficient colonization by other maternal microbes. Nevertheless, the origins of a substantial portion of the meconium and day-14 gut microbiota remain unclear, indicating potential contributions from other maternal sources (e.g., skin and BM) or environmental microbes (4, 44–46).

The correlation network revealed complex cross-niche and temporal interactions between maternal and infant microbes, particularly emphasizing the potential role of maternal microbes in *Bifidobacterium* colonization in the infant gut. In vaginally delivered infants, low-abundance genera like *Lachnospiraceae_NK4A136_group* of VLF were significantly positively correlated with *Bifidobacterium* in VIF1. Additionally, *Lachnospira* and *[Eubacterium]_xylanophilum_group* in VLF were positively correlated with *Bifidobacterium* in VIF2. These findings suggest that specific maternal gut microbes may regulate infants’ intestinal microenvironment through metabolites like short-chain fatty acids, thereby facilitating *Bifidobacterium* colonization (47–49). *Bifidobacterium* is essential for infant gut health, particularly in immune development and metabolic function (50). However, the negative correlation between *Bifidobacterium* in VLF and that in VIF1 is counterintuitive. This may be associated with the enhanced intestinal barrier function by maternal gut *Bifidobacterium*, which reduces its transfer to infant gut (51). Moreover, complex interactions among maternal microbes may also indirectly influence *Bifidobacterium* colonization in the infant gut (52). Notably, three genera of VTP showed significant positive correlations with *Bifidobacterium* in VIF1, suggesting a potential role for placental microbes in early infant gut colonization (16). In cesarean-delivered infants, the correlation network between CTP and CIF1 indicated that low-abundance genera were centrally located and closely interconnected, emphasizing their significance in maternal-infant microbial interactions. These low-abundance genera may promote intestinal health by maintaining microbial ecological balance (53). Additionally, 20 genera in CTP were significantly correlated with *Bifidobacterium* in CIF1, with 18 showing positive correlations. This suggests that placental microbes may influence infant gut microenvironment through metabolic products or immune modulation, thereby enhancing *Bifidobacterium* colonization (54, 55). Similarly, in the correlation network between CIF2 and CTF, significant associations were demonstrated, despite the relatively small contribution of CTF. Some genera of CTF were correlated both positively and negatively with *Bifidobacterium* in CIF2, suggesting that maternal microbiota may regulate intestinal immunity and influence the composition and function of infant gut microbiota (56). In conclusion, our study indicates that maternal microbiota may play a pivotal role in the colonization of early infant gut microbiota. Future research should further elucidate the intricate interactions between maternal and infant microbes to better understand their long-term impact on infant health and development.

Our study demonstrated a certain extent of shared species between the placental microbiota and infant meconium microbiota, particularly anaerobic or facultative anaerobes such as *B. longum* and *Escherichia coli*. The existence of these commensal microbes indicates that placental microbes may be transferred to the fetus through the maternal-fetal interface, in line with previous research (16). Given that these microbes are difficult to survive in the environment, we hypothesize that a certain quantity of commensal microbes indeed exists in the placenta. However, compared with the study by Aagaard et al., the relative abundance of *Escherichia coli* in our placental samples was relatively low, suggesting that placental microbiota may exhibit certain diversity and complexity due to factors such as differences in research methods and sample variation (54). Nonetheless, our data support the idea that the placenta is not a sterile environment but harbors a diverse microbial community (16, 54). Moreover, among the shared species, common environmental microbes like *P. azotoformans* and *R. pickettii* show high relative abundances in placental microbiota (57, 58). The occurrence of these species may be related to surgical operations or hospital environmental exposure, making the possibility of contamination notable. Moreover, it has been noted that *Ralstonia* is one of the dominant genera in the placenta (59), while *Pseudomonas* is frequently detected in oral microbiota of pregnant women and hyperglycemic individuals with periodontitis, suggesting that these microbes may enter placenta via bloodstream (21, 60, 61). The oral microbiota is also regarded as a potential source of placental microbes, further supporting the hypothesis of the presence of specific microbiota in the placenta (54). In addition, the placenta might harbor certain functional microorganisms, such as *Rothia*, *Staphylococcus,* and *Bacteroides*, which may perform immunomodulatory functions in the local placental environment (55, 62). In conclusion, while the potential for environmental contamination cannot be ignored, we consider that some microorganisms in placenta may have potential biological functions. Future research should further explore the biological significance of placental microbiota and its role in maternal-fetal microbial transmission.

The analysis of shared genera between the meconium and infant day-14 gut microbiota elucidates the colonization process of early infant gut microbiota. Regardless of the delivery mode, both the meconium and feces from 14-day-old infants contain a variety of important commensal bacteria, and their colonization is vital for gut health. Over time, in vaginally delivered infants, *Escherichia-Shigella* maintained a high abundance, *Klebsiella* decreased significantly, and *Enterococcus* exhibited an increasing trend. By contrast, in cesarean-delivered infants, the abundance of *Escherichia-Shigella* increased significantly, while facultative anaerobes such as *Enterococcus* gradually decreased. These differences might reflect the influence of the delivery mode on the composition of early infant gut microbiota. Nevertheless, anaerobes such as *Clostridium_sensu_stricto_1* and *Bifidobacterium* increased significantly in both types of infants, aligning with physiological changes during gut development and consistent with previous research findings (63). These results indicate that although the delivery mode affects the composition of early gut microbiota, the colonization of anaerobes is possibly a common trend in the microbial development of infants. Notably, beneficial microbes like *Bifidobacterium*, which possess metabolic and immune regulatory functions (50), increased significantly by day 14, potentially suggesting a transition towards a healthier intestinal environment. This favorable transformation is further emphasized by the significant increase in *Bacteroides*, especially in vaginally delivered infants (32). However, future studies should conduct long-term follow-up to further explore the developmental trends of the gut microbiota and the long-term impacts of dietary and other factors on its regulation.

Despite collecting numerous samples from diverse maternal ecological niches, there remains a lack of comprehensive explanation for the origins of infant gut microbes. The lack of late-pregnancy samples (such as at 32 weeks) from women who delivered via cesarean section might cause the omission of a critical period for microbial transmission, thus neglecting the importance of sampling timing and other ecological niches. Additionally, although 16S rDNA sequencing provides valuable taxonomic information at the genus and species levels, its resolution is limited at the strain level. Future studies employing high-resolution techniques like whole-genome sequencing are expected to more comprehensively reveal the mechanisms of mother-infant microbial transmission (64). Furthermore, our study was limited by a relatively small sample size. Future research should increase the sample size and systematically consider the optimal sampling times and a broader range of potential microbial sources, such as other family members, maternal diet, and infant feeding practices (4), to more comprehensively explore the factors influencing the development of early infant gut microbiota.

In conclusion, this study offers valuable insights into the origins and dynamic changes of gut microbiota during early infancy through 16S rDNA sequencing, Source Tracker analysis, and other bioinformatics methods. The results demonstrated that maternal gut microbiota served as the predominant source for early gut microbiota of vaginally delivered infants, commencing its colonization within the fetus as early as 32 weeks of gestation. In contrast, the contribution of maternal vaginal microbiota to early infant gut microbiota was quite limited. Additionally, placental microbiota also proved to be a significant source of fetal gut microbiota. These discoveries enhance our understanding of the mechanisms involved in the formation of gut microbiota during early infancy and suggest novel strategies for infant health care, especially in promoting the colonization of beneficial microbes. Overall, this research lays the groundwork for future interventions aimed at fostering healthy infant development.

## MATERIALS AND METHODS

### Subjects

Subjects were recruited from the First Affiliated Hospital of Jinan University. Pregnant women with pregnancy complications, multiple pregnancies, preterm birth, chronic medical conditions, and use of antibiotics or probiotics during pregnancy were excluded based on the study design. A total of 26 pregnant women participated in the study, comprizing 17 vaginal deliveries and 9 cesarean deliveries. Their infants also participated in the study. Written informed consent was obtained from all participating women, and informed consent was also provided by the mothers on behalf of their infants. Detailed clinical information about the participants can be found in Table 1. This study was approved by the Medical Ethics Committee of the First Affiliated Hospital of Jinan University and conducted in accordance with the regulations of the Declaration of Helsinki.

### Sample collection

Fecal, oral, and vaginal samples were collected during the third trimester (32 weeks) and at full term before the onset of labor. Following delivery, placental samples, infant meconium samples on the first day of life and stool samples on day 14 of life, as well as BM on day 14 postpartum, were obtained. Stool samples were collected by trained professionals using sterile samplers and special specimen boxes. Vaginal samples were collected using sterile vaginal swabs and placed in special sterile specimen boxes. Prior to oral specimen collection, pregnant women refrained from eating or brushing their teeth. Subsequently, professional staff members rotated a sterile cotton swab back and forth at least 10 times to collect oral samples by wiping the teeth, gums, saliva, and buccal mucosa, and then placed the swab into sterile specimen boxes. After delivery, placental samples were aseptically collected by trained professionals. Within 30 minutes of delivery, 1 cm^3^ of placental tissue was obtained at a distance of 2 cm from the umbilical cord root and 1 cm deep, while avoiding necrotic and calcified areas on the maternal surface of the placenta. The collected tissue was then placed into a sterile specimen box. Before BM collection, the mother cleaned the skin around the nipple and areola as well as her hands with soap and water, donned sterile gloves, and expressed BM directly into a sterile tube. A total of 186 samples were ultimately gathered (Table S1). In addition, the samples were named based on the time of sampling, sample type, and mode of delivery. For maternal samples, the first letter denotes the mode of delivery (V for vaginal delivery, C for cesarean delivery), the second letter represents the time of sampling (L for late pregnancy, T for term pregnancy), and the third letter indicates sample type (O for oral swab, F for feces, V for vaginal swab, P for placenta). For infant samples: the first letter represents mode of delivery (V for vaginal delivery, C for cesarean delivery), followed by I to denote infants and F to indicate feces. The time points for sampling were designated as 1 and 2, corresponding to the meconium sample and the stool sample collected on the 14th day after birth, respectively. Following collection, all samples were transported at low temperature and stored in a −80°C refrigerator until testing.

### DNA extraction, PCR amplification, and 16S rDNA sequencing

The genomic DNA was extracted from the samples using CTAB/SDS method, and the concentration and purity of the DNA were assessed through 1% agarose gel. Following this, the samples were diluted to a concentration of 1 ng/µL with sterile water. The V3–V4 hypervariable region of the 16S rDNA gene was amplified using Phusion High-Fidelity PCR Master Mix with GC Buffer and barcoded primers (341F-806R). Subsequently, the PCR products were mixed with an equal volume of 1× loading buffer containing SYB green and subjected to electrophoresis on a 2% agarose gel. PCR products were mixed in equal density ratios. The pooled PCR products were then purified using a Qiagen Gel Extraction Kit (Qiagen, Germany), followed by construction of the sequencing library using TruSeq DNA PCR-Free Library Preparation Kit (Illumina). After quality assessment using the Qubit 2.0 fluorometer, the library underwent sequencing (46).

### Bioinformatics and statistical analysis

Effective Tags were obtained by splicing, filtering, and removing chimeras from the original reads. The Uparse algorithm (Uparse v7.0.1001, http://www.drive5.com/uparse/) was utilized for clustering all Effective Tags with a 97% consistency (Identity) to form OTUs sequence clustering. The SILVA132 database (http://www.arb-silva.de/) was used for species annotation of OTUs.

The statistical analysis and graph drawing were conducted using IBM SPSS 26.0, R-4.3.1, Qiime-1.9.1, and Gephi-0.10.1. To investigate the impact of individual maternal niche bacteria pools on the early infant gut microbiome, Source Tracker was used to trace the bacterial origins in R software. The R script was obtained from https://github.com/danknights/sourcetracker (65). Bioinformatics analyses were carried out on the cloud platform https://magic.novogene.com/. α andβ diversity were calculated using Qiime software, with Shannon and Simpson indices used to assess species richness and evenness for α diversity evaluation. The ggplot2 package in R was utilized to visualize α diversity using box plots. Differences between groups were assessed using the non-parametric Kruskal-Wallis test, and pairwise comparisons were performed using the Wilcoxon rank sum test with Bonferroni correction. The β diversity among microbial communities was assessed using weighted and unweighted_unifrac distance matrices and visualized through PCoA. ANOSIM analysis was conducted with the vegan package in R software to compare variations in microbial community composition across different groups. Bar plots illustrating the relative abundance of species and Venn diagrams were generated using R software. Additionally, Spearman correlation analysis was employed to uncover associations among microbial communities, with a significance threshold of *P* < 0.05 and an absolute value of the correlation coefficient (|*r*|) >0.6. The microbial correlation network was visualized using the psych and reshape2 packages in R software, as well as Gephi software. LEfSe was utilized to identify differential biomarkers among groups. In the analysis of clinical data, measurement data were presented as mean ± standard deviation (SD). The comparison of sample means was conducted using Student’s *t*-test, while the Wilcoxon rank-sum test was used for non-normally distributed data. Count data were presented as percentages, and Fisher’s exact test was used to compare groups. A *P* value < 0.05 was considered statistically significant.

## Data Availability

The data supporting the findings of this study are openly available in the NCBI database under the accession number PRJNA1105345.

## SUPPLEMENTAL MATERIAL

Tables S1 to S12 spectrum.01775-25-s0001.xlsx Information on sample collection, microbial distribution across different maternal and infant stages and ecological niches, and correlation analyses of cross-niche microbial interaction networks between mothers and infants.

Tables S13 to S16 spectrum.01775-25-s0002.docx Analyses of shared species between placental and meconium microbiota in cesarean and vaginally delivered infants, as well as shared taxa between meconium and day-14 gut microbiota in infants born via different delivery modes.

ASM does not own the copyrights to Supplemental Material that may be linked to, or accessed through, an article. The authors have granted ASM a non-exclusive, world-wide license to publish the Supplemental Material files. Please contact the corresponding author directly for reuse.
