# Supplementary material for: Establishment of the early gut microbiota in vaginally delivered infants: the influence of maternal gut microbiota outweighs vaginal microbiota
Source: Microbiol Spectr. 2025 Aug 12;13(9):e01775-25. doi: 10.1128/spectrum.01775-25 (PMC12403616; doi:10.1128/spectrum.01775-25)
Supplement: Tables S13 to S16 — Analyses of shared species between placental and meconium microbiota in cesarean and vaginally delivered infants, as well as shared taxa between meconium and day-14 gut microbiota in infants born via different delivery modes. [file spectrum.01775-25-s0002.docx]

**Table S13** The shared species between full-term placental microbiota and meconium microbiota of vaginally delivered infants

| **Species** | **VTP (Relative abundance, %)** | **VIF1 (Relative abundance, %)** | **Common human symbiotic bacteria** | **Existing environment** | **Aerotolerance** |
| --- | --- | --- | --- | --- | --- |
| *Pseudomonas azotoformans* | 64.1 | <0.1 | No | Soil, water | Aerobic |
| *Ralstonia pickettii* | 6.7 | 24.3 | No | Water, soil | Aerobic |
| *Rothia mucilaginosa* | 0.1 | 0.1 | Yes | Oral cavity, upper respiratory tract | Facultative anaerobic |
| *Haemophilus parainfluenzae* | 0.1 | <0.1 | Yes | Upper respiratory tract | Facultative anaerobic |
| *Escherichia coli* | <0.1 | 32.2 | Yes | Intestine | Facultative anaerobic |
| *Klebsiella aerogenes* | <0.1 | 10.1 | No | Hospital environment, water, soil | Facultative anaerobic |
| *Bifidobacterium longum* | <0.1 | 0.8 | Yes | Intestine | Anaerobic |
| *Bacteroides plebeius* | <0.1 | 0.6 | Yes | Intestine | Anaerobic |
| *Staphylococcus caprae* | <0.1 | 0.5 | No | Human and animal skin, goat milk | Facultative anaerobic |
| *Brevundimonas vesicularis* | <0.1 | 0.4 | No | Water, soil | Aerobic |
| *Enterococcus faecalis* | <0.1 | 0.4 | Yes | Intestine | Facultative anaerobic |
| *Bacteroides vulgatus* | <0.1 | 0.3 | Yes | Intestine | Anaerobic |
| *Cutibacterium acnes* | <0.1 | 0.1 | Yes | Skin | Anaerobic |
| *Lactococcus lactis* | <0.1 | 0.1 | No | Dairy products | Facultative anaerobic |

The aerotolerance of species is primarily determined using the eighth edition of Bergey's Manual of Determinative Bacteriology and the Bacterial Diversity Metadatabase. Abbreviation: VTP, full-term placental microbiota of vaginal delivery; VIF1, meconium microbiota of vaginally delivered infants.

**Table S14** The shared species between full-term placental microbiota and meconium microbiota of cesarean-section infants

| **Species** | **CTP (Relative abundance, %)** | **CIF1 (Relative abundance, %)** | **Common human symbiotic bacteria** | **Existing environment** | **Aerotolerance** |
| --- | --- | --- | --- | --- | --- |
| *Pseudomonas azotoformans* | 22.6 | 32.7 | No | Soil, water | Aerobic |
| *Ralstonia pickettii* | 1.6 | 3.1 | No | Water, soil | Aerobic |
| *Escherichia coli* | 0.9 | 1.1 | Yes | Intestine | Facultative anaerobic |
| *Staphylococcus caprae* | 0.6 | 7.1 | No | Human and animal skin, goat milk | Facultative anaerobic |
| *Faecalibacterium prausnitzii* | 0.5 | 0.1 | Yes | Intestine | Anaerobic |
| *Bacteroides xylanisolvens* | 0.4 | <0.1 | Yes | Intestine | Anaerobic |
| *Bacteroides vulgatus* | 0.2 | <0.1 | Yes | Intestine | Anaerobic |
| *Romboutsia ilealis* | 0.2 | 0.2 | No | Animal intestine | Anaerobic |
| *Akkermansia muciniphila* | 0.2 | 0.1 | Yes | Intestine | Anaerobic |
| *Bacteroides uniformis* | 0.2 | <0.1 | Yes | Intestine | Anaerobic |
| *Methylobacterium komagatae* | 0.2 | <0.1 | No | Soil | Aerobic |
| *Bacteroides plebeius* | 0.2 | <0.1 | Yes | Intestine | Anaerobic |
| *Bacteroides fragilis* | 0.1 | <0.1 | Yes | Intestine | Anaerobic |
| *Vibrio campbellii* | 0.1 | <0.1 | No | Seawater | Aerobic or facultative anaerobic |
| *Bacillus velezensis* | 0.1 | <0.1 | No | Soil | Aerobic |
| *Pantoea cypripedii* | 0.1 | 0.3 | No | Plant, soil | Facultative anaerobic |
| *Delftia tsuruhatensis* | <0.1 | 0.1 | No | Water, soil | Aerobic |
| *Acinetobacter baumannii* | <0.1 | 5.3 | No | Hospital environment, water, soil | Aerobic |
| *Enterococcus faecalis* | <0.1 | 9.5 | Yes | Intestine | Facultative anaerobic |
| *Corynebacterium aurimucosum* | <0.1 | 0.3 | Yes | Skin, mucosa | Aerobic or facultative anaerobic |

The aerotolerance of species is primarily determined using the eighth edition of Bergey's Manual of Determinative Bacteriology and the Bacterial Diversity Metadatabase. Abbreviation: CTP, full-term placental microbiota of cesarean section; CIF1, meconium microbiota of cesarean-section infants.

**Table S15** The shared genera between meconium microbiota and day-14 gut microbiota of vaginally delivered infants

| **Genus** | **VIF1 (Relative abundance, %)** | **VIF2 (Relative abundance, %)** | **Human symbiotic bacteria** | **Existing environment** | **Aerotolerance** | **Function** |
| --- | --- | --- | --- | --- | --- | --- |
| *Escherichia-Shigella* | 32.2 | 27.2 | Yes | Intestine | Facultative anaerobic | Contributes to gut health. Some strains can cause intestinal infections. (1) |
| *Ralstonia* | 24.3 | <0.1 | No | Soil, water | Aerobic | Plant pathogen; potential nosocomial pathogen.(2, 3) |
| *Streptococcus* | 15.4 | 6.1 | Yes | Oral cavity, throat, intestine, vagina | Facultative anaerobic | Some strains cause pneumonia and oral diseases; some strains are beneficial to health.(4-6) |
| *Klebsiella* | 10.1 | 2.4 | Yes | Intestine, respiratory tract | Facultative anaerobic | Opportunistic pathogen, can cause respiratory and urinary infections.(7, 8) |
| *Acinetobacter* | 7 | 0.4 | No | Soil, water | Aerobic | Opportunistic human pathogen, often causes infections in immunocompromised individuals.(9) |
| *Bifidobacterium* | 3.2 | 25.5 | Yes | Intestine | Anaerobic | Promotes gut health, involved in metabolism and immune regulation.(10) |
| *Pantoea* | 1.2 | 0.7 | No | Plants, water, soil | Facultative anaerobic | Beneficial to plant growth, some strains cause plant and human infections.(11) (12) |
| *Bacteroides* | 1.1 | 11.4 | Yes | Intestine | Anaerobic | Ferments dietary fibers, produces short-chain fatty acids.(13) |
| *Halomonas* | 1 | 0.1 | No | Saline environments | Aerobic | Found in high-salt environments, has potential biotechnological applications.(14) |
| *Enterococcus* | 0.5 | 3.4 | Yes | Intestine | Facultative anaerobic | Normal gut flora, but some strains can cause infections such as urinary tract infections.(15) |
| *Staphylococcus* | 0.5 | 1.6 | Yes | Skin, mucosa | Facultative anaerobic | Common pathogen, can cause skin and other infections.(16) |
| *Pectobacterium* | 0.2 | 7.4 | No | Plants, soil | Facultative anaerobic | Mainly plant pathogen.(17) |
| *Clostridium_sensu_stricto_1* | 0.1 | 2.9 | Yes | Water, soil, intestine | Anaerobic | Participates in fermentation, produces short-chain fatty acids, some strains may cause infections.(18, 19) |
| *Parabacteroides* | 0.1 | 1.9 | Yes | Intestine | Anaerobic | Involved in metabolism and immune regulation.(20) |
| *Collinsella* | <0.1 | 2.3 | Yes | Intestine | Anaerobic | Involved in carbohydrate metabolism, produces short-chain fatty acids.(21) |

The aerotolerance of species is primarily determined using the eighth edition of Bergey's Manual of Determinative Bacteriology and the Bacterial Diversity Metadatabase. Abbreviation: VIF1, meconium microbiota of vaginally delivered infants; VIF2, gut microbiota of 14-day-old vaginally delivered infants.

**Table S16** The shared genera between meconium microbiota and day-14 gut microbiota of infants delivered by cesarean section

| **Genus** | **CIF1 (Relative abundance, %)** | **CIF2 (Relative abundance, %)** | **Human symbiotic bacteria** | **Existing environment** | **Aerotolerance** | **Function** |
| --- | --- | --- | --- | --- | --- | --- |
| *Acinetobacter* | 8.9 | 0.4 | No | Soil, Water | Aerobic | Opportunistic human pathogen, often causes infections in immunocompromised individuals.(9) |
| *Enterococcus* | 9.5 | 0.2 | Yes | Intestine | Facultative anaerobic | Normal gut flora, but some strains can cause infections such as urinary tract infections. (15) |
| *Escherichia-Shigella* | 1.1 | 24.8 | Yes | Intestine | Facultative anaerobic | Contributes to gut health. Some strains can cause intestinal infections.(1) |
| *Staphylococcus* | 16.2 | 0.3 | Yes | Skin, mucosa | Facultative anaerobic | Common pathogen, can cause skin and other infections.(16) |
| *Streptococcus* | 5 | 18.5 | Yes | Oral cavity, throat, intestine, vagina | Facultative anaerobic | Some strains cause pneumonia and oral diseases; some strains are beneficial to health.(4-6) |
| *Bifidobacterium* | 0.2 | 33.2 | Yes | Intestine | Anaerobic | Promotes gut health, involved in metabolism and immune regulation.(10) |
| *Clostridium_sensu_stricto_1* | 0.1 | 9 | Yes | Water, soil, intestine | Anaerobic | Participates in fermentation, produces short-chain fatty acids, some strains may cause infections.(18, 19) |
| *Pantoea* | 0.4 | 1.5 | No | Plants, water, soil | Facultative anaerobic | Beneficial to plant growth, some strains cause plant and human infections.(11, 12) |
| *Rothia* | ＜0.1 | 1.3 | Yes | Oral cavity, throat | Aerobic | Involved in oral health, some species can cause infections.(22, 23) |
| *Veillonella* | 0.1 | 6.3 | Yes | Oral cavity, intestine, respiratory tract | Anaerobic | Involved in lactate metabolism, converts it into short-chain fatty acids, beneficial for gut health.(24) |

The aerotolerance of species is primarily determined using the eighth edition of Bergey's Manual of Determinative Bacteriology and the Bacterial Diversity Metadatabase. Abbreviation: CIF1, meconium microbiota of cesarean-section infants; CIF2, gut microbiota of 14-day-old infants delivered by cesarean section.

**References**

1. Moreira de Gouveia MI, Bernalier-Donadille A, Jubelin G. 2024. Enterobacteriaceae in the Human Gut: Dynamics and Ecological Roles in Health and Disease. Biology (Basel) 13:142.

2. Rivera-Zuluaga K, Hiles R, Barua P, Caldwell D, Iyer-Pascuzzi AS. 2023. Getting to the root of Ralstonia invasion. Semin Cell Dev Biol 148-149:3-12.

3. Satirer O, Henes JC, Doring M, Lesk T, Benseler S, Kuemmerle-Deschner JB. 2024. Autologous haematopoiesis stem cell transplantation (AHSCT) for treatment-refractory autoimmune diseases in children. RMD Open 10:e004381.

4. Ye D, Liu Y, Li J, Zhou J, Cao J, Wu Y, Wang X, Fang Y, Ye X, Zou J, Ma Q. 2025. Competitive dynamics and balance between Streptococcus mutans and commensal streptococci in oral microecology. Crit Rev Microbiol 51:532-543.

5. Xu Y, Wang J, Qin X, Liu J. 2024. Advances in the pathogenesis and treatment of pneumococcal meningitis. Virulence 15:2387180.

6. Gao ZY, Cui Z, Yan YQ, Ning LJ, Wang ZH, Hong J. 2021. Microbe-based management for colorectal cancer. Chin Med J (Engl) 134:2922-2930.

7. Timm MR, Russell SK, Hultgren SJ. 2025. Urinary tract infections: pathogenesis, host susceptibility and emerging therapeutics. Nat Rev Microbiol 23:72-86.

8. Douradinha B. 2024. Exploring the journey: A comprehensive review of vaccine development against Klebsiella pneumoniae. Microbiol Res 287:127837.

9. Breisch J, Schumm C, Poehlein A, Daniel R, Averhoff B. 2022. The carnitine degradation pathway of Acinetobacter baumannii and its role in virulence. Environ Microbiol 24:4437-4448.

10. Cheng Y, Liu J, Ling Z. 2022. Short-chain fatty acids-producing probiotics: A novel source of psychobiotics. Crit Rev Food Sci Nutr 62:7929-7959.

11. Lv L, Luo J, Ahmed T, Zaki HEM, Tian Y, Shahid MS, Chen J, Li B. 2022. Beneficial Effect and Potential Risk of Pantoea on Rice Production. Plants (Basel) 11:2608.

12. Cruz AA, Cabeo M, Duran-Viseras A, Sampedro I, Llamas I. 2024. Interference of AHL signal production in the phytophatogen Pantoea agglomerans as a sustainable biological strategy to reduce its virulence. Microbiol Res 285:127781.

13. Xu T, Wu X, Liu J, Sun J, Wang X, Fan G, Meng X, Zhang J, Zhang Y. 2022. The regulatory roles of dietary fibers on host health via gut microbiota-derived short chain fatty acids. Curr Opin Pharmacol 62:36-42.

14. Biswas J, Jana SK, Mandal S. 2023. Biotechnological impacts of Halomonas: a promising cell factory for industrially relevant biomolecules. Biotechnol Genet Eng Rev 39:348-377.

15. Fiore E, Van Tyne D, Gilmore MS. 2019. Pathogenicity of Enterococci. Microbiol Spectr 7.

16. Burke O, Zeden MS, O'Gara JP. 2024. The pathogenicity and virulence of the opportunistic pathogen Staphylococcus epidermidis. Virulence 15:2359483.

17. Gorshkov V, Parfirova O. 2023. Host plant physiological transformation and microbial population heterogeneity as important determinants of the Soft Rot Pectobacteriaceae-plant interactions. Semin Cell Dev Biol 148-149:33-41.

18. Tsai CC, Jette S, Tremlett H. 2024. Disease-modifying therapies used to treat multiple sclerosis and the gut microbiome: a systematic review. J Neurol 271:1108-1123.

19. Chen YY, Chen SY, Chang HY, Liu YC, Chuang BF, Yen GC. 2024. Phyllanthus emblica L. polysaccharides ameliorate colitis via microbiota modulation and dual inhibition of the RAGE/NF-kappaB and MAPKs signaling pathways in rats. Int J Biol Macromol 258:129043.

20. Hasain Z, Mokhtar NM, Kamaruddin NA, Mohamed Ismail NA, Razalli NH, Gnanou JV, Raja Ali RA. 2020. Gut Microbiota and Gestational Diabetes Mellitus: A Review of Host-Gut Microbiota Interactions and Their Therapeutic Potential. Front Cell Infect Microbiol 10:188.

21. Yan J, Xiao L, Feng D, Chen B, Yang T, Tong B, Luo R, Wang Y, Chen J. 2023. Vitamin A deficiency suppresses CEACAM1 to impair colonic epithelial barrier function via downregulating microbial-derived short-chain fatty acids. Genes Dis 11:1066-1081.

22. Rosier BT, Takahashi N, Zaura E, Krom BP, MartInez-Espinosa RM, van Breda SGJ, Marsh PD, Mira A. 2022. The Importance of Nitrate Reduction for Oral Health. J Dent Res 101:887-897.

23. Espinoza-Candelaria GJ, Mamauag E, Bukowinski A, Aldewereld Z. 2024. Successful Treatment of a Fulminant Rothia mucilaginosa Central Nervous System Infection in an Adolescent With T-Cell Acute Lymphoblastic Leukemia. Pediatr Infect Dis J 43:e255-e256.

24. Hung JH, Zhang SM, Huang SL. 2024. Nitrate promotes the growth and the production of short-chain fatty acids and tryptophan from commensal anaerobe Veillonella dispar in the lactate-deficient environment by facilitating the catabolism of glutamate and aspartate. Appl Environ Microbiol 90:e0114824.
